# Do not let perfect be the enemy of good: the current reality of *Bordetella* testing

**DOI:** 10.1128/spectrum.02210-24

**Published:** 2024-12-09

**Authors:** Rebecca Yee

**Affiliations:** 1 Department of Pathology, George Washington University School of Medicine and Health Sciences, Washington, DC, USA; MultiCare Health System, Tacoma, Washington, USA

**Keywords:** *Bordetella* real time-PCR, non-pertussis *Bordetella*, pertussis diagnostics, pertussis surveillance

## Abstract

Diagnosis of *Bordetella pertussis* is made by using polymerase chain reaction (PCR) to detect insertion sequence 481 (IS*481*). However, IS*481* is found in both *B. pertussis* and *Bordetella holmesii*. In a recent study, Cole et al. (Microbiol Spectr 12:e00783-24. https://doi.org/10.1128/spectrum.00783-24) compared the accuracy of assays performed at two different commercial laboratories that used IS*481* as a molecular target against a multiplex PCR assay performed at the Centers for Disease Control and Prevention (CDC) that can differentiate the different *Bordetella* species. The results demonstrated that the overall agreement among the assays from the commercial laboratories and the CDC was 85%. Only a small percentage (1.4%) of specimens positive for IS*481* were identified as *B. holmesii*. This study suggests that PCR tests using IS*481* as a target provide reliable diagnosis for pertussis. However, end users should recognize the limitations of PCR testing for pertussis including the inability to differentiate and monitor transmission of non-pertussis *Bordetella* species.

## COMMENTARY

*Bordetella pertussis* causes the highly contagious respiratory illness known as the “whooping cough” which continues to be endemic in the United States (1). Only infections with *B. pertussis* are a reportable condition in the United States so the burden of non-pertussis *Bordetella* species (*Bordetella parapertussis*, *Bordetella holmesii*, and *Bordetella bronchiseptica*) is not well understood. However, non-pertussis species have been reported to cause severe illnesses in infants and immunocompromised individuals (2). Thus, diagnosing infections with *Bordetella* species is critical for controlling outbreaks, guiding treatment, and supporting public health interventions.

Detection of *Bordetella* is usually done using polymerase chain reaction (PCR) given its higher sensitivity and specificity over methods such as direct fluorescent antibody staining or culture. PCR is two- to sixfold more sensitive than culture, although performance may vary depending on the patient’s age, vaccination status, and duration of symptoms (3). Multicopy genomic targets used in *Bordetella* PCR assays include (i) the insertion sequence 481 (IS*481*), present in *B. pertussis*, *B. holmesii*, and very rarely, *B. bronchiseptica*, (ii) the insertion sequence 1001 (IS*1001*), present in *B. parapertussis* and *B. bronchiseptica*, and (iii) the insertion sequence 1002 (IS*1002*), present in *B. parapertussis* and *B. pertussis* (4). Sometimes monogenic targets such as hIS*1001*/*recA* and *ptxP* are used for *B. holmesii* and *B. pertussis* detection, respectively (4). Differentiation between *B. pertussis* and the non-pertussis species can be a challenge as the same targets are found in different species of *Bordetella*.

Cole et al. compared the performance of commercial laboratories’ assays for the detection of *Bordetella* species to their assay at the Centers for Disease Control and Prevention (CDC, United States), which is a multiplex PCR assay capable of differentiating the separate species (5). They also assessed the proportion of specimens that were positive for non-pertussis species. This study was performed on 3,984 clinical specimens positive for IS*481* or IS*1001* from two commercial laboratories during 2012–2019.

Agreement of 84.6% was achieved among the commercial and CDC assays. Specifically, the agreement was 87% (3,181/3,663) and 98.4% (305/310) for detection of *B. pertussis/B. holmesii* and *B. parapertussis/B. bronchiseptica*, respectively. Reproducibility of the *C*_*t*_  values depended on the molecular target; reproducibility of the *C*_*t*_ values was higher for IS*481* than IS*1001*.

The breakdown of the *Bordetella* species detected was as follows: 78% *B. pertussis*, *7*% *B. parapertussis*, 1.4% *B. holmesii*, and 0.1% with both *B. pertussis* and  *B. holmesii*. Specimens with *B. holmesii* were primarily from patients in California (43.6%). The average positivity rate of *B. holmesii*  was 1.3% per year, a rate reported in other countries (6). However, it is fair to say that active, ongoing, surveillance either through species-targeted, PCR-based methods or next-generation sequencing approaches may be necessary to capture a more accurate distribution of *Bordetella* species rather than testing specimens retrospectively as was done here.

Molecular methods like PCR are sensitive, have a rapid turnaround time, and can be high throughput. Despite the advantages, it is paramount that users of these tests acknowledge the limitations and variability of molecular testing for clinical care. The premise behind the manuscript by Cole et al. stems from uncovering the potential diagnostic inaccuracies due to the molecular target used for pertussis testing. However, discordances can also be due to the testing process from DNA extraction to amplification, specimen stability, and the assay’s limit of detection. This is especially important for specimens with low burden of organisms associated with lower severity of disease and specimen collection variability. As shown in the findings from Cole et al., discordant results were from specimens with *C*_*t*_ values between 35 and 40 (35 ≥ *C*_*t*_  < 40) which is on the upper end of the limit of detection. Additionally, cross-reactivity and specificity issues may arise. An assay’s performance characteristics depend on the population’s true prevalence of disease, undermining the importance of evaluating negative specimens, which this study did not include. To eliminate confounding factors such as specimen instability, a head-to-head comparison should be performed with fresh specimens, instead of specimens used in this study.

The advent of FDA-approved molecular panels allows clinical microbiology laboratories to offer rapid, sensitive PCR testing as a diagnostic tool. Currently, several commercially available, syndromic respiratory viral panels include *Bordetella* as a target for detection and the genetic marker(s) used on these panels may be IS*481* or the *ptxP* gene, depending on the manufacturer. Variable performance (accuracy of 66–100%) has been reported and can be attributed to the molecular target and/or the bacterial burden in the specimen (7). It is important for clinical laboratories to acknowledge the variability in performance prior to implementing the tests.

The increase in pertussis cases has been linked to several factors such as waning immunity from acellular pertussis vaccines, greater awareness among healthcare providers resulting in more testing, and genetic variations of circulating *B. pertussis* strains (2, 8, 9). While PCR testing is a sensitive approach to detect pertussis, using the IS*481* insertion sequence as a target has the potential to misclassify *B. holmesii* as *B. pertussis* or to detect DNA from pertussis vaccination, warranting clinicians to interpret PCR results with caution. The future of *Bordetella* diagnostics will likely focus on improving the precision of diagnosis, differentiating between *Bordetella* species, integrating genomic surveillance methodologies to allow tracking of genetic variants, and enhancing public health responses to aid interventions and vaccination strategies. In this recent benchmarking study, Cole et al. demonstrated that commercial laboratories using IS*481*-testing offer accurate diagnosis of *B. pertussis* and *B. parapertussis* infections in the United States, with only a minor subset being positive for *B. holmesii*. Their findings reassure our trust in the current PCR tests available for pertussis testing while we wait for exciting diagnostic developments in the future.
